# Medical Commencement

**Published:** 1848-05

**Authors:** 


### THE WESTERN JOURNAL.

THIRD SERIES.—VOL. I.—NO V.

LOUISVILLE, MAY 1, 1848.

### MEDICAL COMMENCEMENT.

The second annual commencement in the University of Louisville—the eleventh in the school—was held on Wednesday evening, the 8th of March, on which occasion the degree of M. D. was conferred on ninety-four of the alumni of the institution. Professor Drake delivered the valedictory address to the graduates.

